# Fluorescence guidance in skull base surgery: Applications and limitations – A systematic review

**DOI:** 10.1016/j.bas.2024.103328

**Published:** 2024-08-29

**Authors:** Eric Suero Molina, Michael Bruneau, Gilles Reuter, Mostafa Shahein, Luigi M. Cavallo, Roy T. Daniel, Ekkehard M. Kasper, Sebastien Froelich, Emanuel Jouanneau, Romain Manet, Mahmoud Messerer, Diego Mazzatenta, Torstein R. Meling, Pierre-Hugues Roche, Henry WS. Schroeder, Marcos Tatagiba, Massimiliano Visocchi, Daniel M. Prevedello, Walter Stummer, Jan F. Cornelius, EANS Skull Base Section

**Affiliations:** a Department of Neurosurgery, University Hospital of Münster, Münster, Germany; b Macquarie Medical School, Faculty of Medicine, Health and Human Sciences, Macquarie University, Sydney, Australia; c Department of Neurosurgery, Universitair Ziekenhuis Brussel, Vrije Universiteit Brussel (VUB), Brussels, Belgium; d Department of Neurosurgery, University Hospital of Liège, Liège, Belgium; e Department of Neurosurgery, Mansoura University, Egypt; f Department of Neurosciences and Reproductive and Dental Sciences, Division of Neurosurgery, Federico II University of Naples, Policlinico Federico II University Hospital, Italy; g Department of Neurosurgery, Department of Neuroscience, Centre Hospitalier Universitaire Vaudois, University Hospital Lausanne, Switzerland; h Department of Neurosurgery, Boston University Medical School, MA and Steward Medical Group, Brighton, MA/USA McMaster University Faculty of Health Sciences, Hamilton, ON, Canada; i Department of Neurosurgery, Lariboisière Hospital, Université Paris Diderot, Paris, France; j Department of Neurosurgery, Hôpital Neurologique Pierre Wertheimer, Lyon, France; k Department of Neurosurgery, Neurological Sciences Institut IRCCS, Bologna, Italy; l Department of Neurosurgery, The National Hospital, Rigshospitalet, Copenhagen, Denmark; m Department of Neurosurgery, Aix-Marseille Université, Assistance Publique-Hôpitaux de Marseille, Hôpital Nord, Marseille, France; n Department of Neurosurgery, University Medicine Greifswald, Germany; o Department of Neurosurgery, University Hospital Tübingen, Tübingen, Germany; p Department of Neurosurgery, Institute of Neurosurgery Catholic University of Rome, Italy; q Deparmtent of Neurosurgery, The Ohio State University College of Medicine, Columbus, OH, USA; r Department of Neurosurgery, University Hospital of Düsseldorf, Heinrich Heine University, Düsseldorf, Germany

**Keywords:** 5-ALA, Fluorescein, Indocyanine-green, Fluorescence-guided resection, Skull base tumors, Endoscopic endonasal surgery

## Abstract

**Introduction:**

Intraoperative fluorescence guidance is a well-established surgical adjunct in high-grade glioma surgery. In contrast, the clinical use of such dyes and technology has been scarcely reported in skull base surgery.

**Research question:**

We aimed to systematically review the clinical applications of different fluorophores in both open and endonasal skull base surgery.

**Material and methods:**

We performed a systematic review and discussed the current literature on fluorescence guidance in skull base surgery.

**Results:**

After a comprehensive literature search, 77 articles on skull base fluorescence guidance were evaluated. A qualitative analysis of the articles is presented, discussing clinical indications and current controversies. The use of intrathecal fluorescein was the most frequently reported in the literature. Beyond that, 5-ALA and ICG were two other fluorescent dyes most extensively discussed, with some experimental fluorophore applications in skull base surgery.

**Discussion and conclusion:**

Intraoperative fluorescence imaging can serve as an adjunct technology in skull base surgery. The scope of initial indications of these fluorophores has expanded beyond malignant glioma resection alone. We discuss current use and controversies and present an extensive overview of additional indications for fluorescence imaging in skull base pathologies. Further quantitative studies will be needed in the future, focusing on tissue selectivity and time-dependency of the different fluorophores currently commercially available, as well as the development of new compounds to expand applications and facilitate skull base surgeries.

## Highlights

• This review highlights the lack of standardization in fluorescence use in skull base surgeries, summarizing current literature and practices.
• This review summarizes current literature on fluorescent dyes in skull base surgery and outlines future research directions.
• ICG's deep tissue penetration and minimal autofluorescence interference provide clear visualization of vascular structures and tumors in the skull base, reducing light scattering.
• 5-ALA aids skull base tumor resection, enhancing fluorescence for better outcomes and identifying bony infiltration in meningiomas.
• Fluorescein detection of CSF leaks is the most reported application of a fluorophore in skull base surgery.

## 1 Introduction

Intraoperative fluorescence guidance enhances real-time visualization of tumor tissue, aiding in maximizing resection. To date, fluorescence-guided surgery (FGS) has revolutionized intraoperative visualization of primary intraparenchymal central nerve system tumors, predominantly of malignant glioma types (Stummer et al., 2006; Schipmann et al., 2020; Suero Molina et al., 2017, 2019; Stummer and Suero, 2017; Della Puppa et al., 2013; Cornelius et al., 2013a; Widhalm et al., 2013; Hefti et al., 2008; Xiang et al., 2018; Michael et al., 2019; Nabavi et al., 2009; Hamamcioglu et al., 2016; Hadjipanayis et al., 2015) and it has been successfully applied to assess vessel patency in the surgical care of cerebrovascular malformations (Raabe et al., 2003; Zhao et al., 2019; Nickele et al., 2019). Before its widespread adoption in neurosurgery, FGS demonstrated its utility for clinical diagnosis and disease treatment had been demonstrated in numerous clinical disciplines, including urology, dermatology, and ophthalmology (Georges et al., 2019).

In neurooncology, intraoperative fluorescence guidance has emerged as a pivotal area of research, providing valuable insights into tumor invasion and biology (Stummer et al., 2006). With dedicated advanced technology microscopes, fluorophore may become observable through the conventional oculars of a standard microscope or via image processing on monitors. Fluorescence intensity is used as a common measure to characterize and describe the quality of the optic signal, while quantitative or semi-quantitative methods (e.g., spectroscopy) are subject of current research (Walke et al., 2023; Cornelius et al., 2017a; Black et al., 2021; Knipps et al., 2017, 2019; [Bibr bib66][Bibr bib135], Suero Molina et al., 2023). Cameras to detect fluorescent dyes have also been integrated into modern endoscopes and can be employed during different skull base procedures (Muto et al., 2023; Eljamel et al., 2009; Litvack et al., 2012; Verstegen et al., 2016; Lee and Lee, 2022; Shahein et al., 2018, 2020).

The present article focuses on three fluorescent dyes predominantly used in current neurosurgical practice: 5-aminolevulinic acid (5-ALA), Fluorescein, and indocyanine green (ICG) (Table 1). Furthermore, we shall give an outlook on the possible applications of other experimental dyes currently investigated in research.

**(Table 1):** Evaluation of different fluorophores and their clinical applications.

Fluorophore	Application	Utility
5-ALA	Chordomas	Time-dependent uptake in chordoma cell lines (U-CH2). Possibility of experimental PDT.
	Meningiomas	Tumor resection. Correlation between fluorescence intensity and histologically specified WHO grade.
	Endoscopic Endonasal Skull Base Surgery	A recent study concludes that except for meningioma, 5-ALA did not help resect pathologies.
Fluorescein	CSF leak	Precisely localizes skull base defects and confirms repair integrity.
	Meningiomas	Tumor delineation and video-angiography for vessel patency assessment.
	Droplet Staining in Endonasal Surgery	Visual indicator for droplet contamination during endonasal surgery in SARS-CoV-2 pandemic.
ICG	Endoscopic Endonasal Skull Base Surgery	Identifies vascular anatomical landmarks (e.g. ICAs) and assesses and monitors patency of vessels. Second window technique can be used to observe selective enhancement in tumor tissue.
	Vascularized Flaps	Evaluates real-time perfusion in nasoseptal and pericranial flaps.
	Meningiomas	Tumor delineation.
	Malignant Neoplasms	Guidance for superselective intra-arterial chemotherapy for skull base malignancies.

5-ALA = 5-aminolevulinic acid, CSF = cerebrospinal fluid, PDT= Photodynamic therapy, ICA=Internal carotid artery, ICG = indocyanine green

Many commercially available fluorescent dyes do not cross the intact blood-brain barrier (BBB) in significant quantities, therefore requiring disruption of the BBB for tissue penetration in suitable concentration. This may occur either in a passive way where fluorophores cross gaps in the BBB, get extravasated and penetrate the brain parenchyma and tumor tissue, or remain as perifocal edema (such as Fluorescein and ICG). Alternatively, the compounds may be actively metabolized by tumor tissue, resulting in a specific accumulation of endogenous fluorescent agents (e.g., 5-ALA leading to the accumulation of fluorescent protoporphyrin IX, PpIX). The latter might also involve a disruption in the BBB. Molecularly targeted agents (e.g., Chlorotoxins) bind specifically to molecules and represent another class of dyes being investigated for tumor applications in current scientific research (Hadjipanayis and Stummer, 2019a; Linsler et al., 2021; Jarmula et al., 2022).

While fluorescence guidance has been widely adopted in glioma surgery, applications in skull base surgery remain scarcely described. We provide a comprehensive overview of the available literature on current applications in skull base surgery in hopes of providing a common ground for further research on fluorescent dyes.

## 2 Methods

### 2.1 Literature search with preferred reporting items for systematic review and meta-analysis (PRISMA)

We conducted an individual search with assistance of the Macquarie University Library for this review in PubMed/Medline and Embase according to the PRISMA statement (Moher et al., 2009, 2010).

We performed our search for articles in the English language published until the 15th of September 2023. The following terms were used in our search algorithm for titles and abstracts using the Boolean operator “and”: “Skull Base” and “fluorescence”, “Skull Base” and “5-ALA”, “Skull Base” and “5-Aminolevulinic acid”, “Skull Base” and “ICG”, Skull Base” and “indocyanine green”, Skull Base” and “fluorescein”. After excluding unsuitable articles by removing duplicates and non-English articles and screening titles and abstracts, we specifically selected studies focusing on intraoperative FGS at the skull base. We also excluded “vascular” articles, focusing on the patency of vessels with ICG. In other terms, strict “vascular” articles were excluded. Retrieved articles were managed with Endnote X9 (Thompson Reuters, Carlsbad, California, USA).

## 3 Results

After application of all inclusion criteria, our literature search yielded 243 articles for review. We then applied exclusion criteria and removed all duplicates (n = 74) and non-English articles (n = 16). After the abstract review, we removed a further set of non-relevant articles (n = 76), which left us with 77 articles for full-text in-depth evaluation. An additional number of pertinent reports were identified by cross-reference check and also included for analysis. The evaluated fluorescent dyes were 5-ALA (Cornelius et al., 2013a, 2014, 2017a, 2017b, 2019; Eljamel et al., 2009; Goryaynov et al., 2019; Soleman et al., 2013; Turcotte et al., 2020; Bekelis et al., 2011; Della Puppa and Scienza, 2013; Della Puppa et al., 2014; Potapov et al., 2016; Micko et al., 2020; Recinos, 2020; Neumann et al., 2016), Fluorescein (Anari et al., 2007; Banu et al., 2014; Zhang et al., 2018; Tabaee et al., 2007; Akcakaya et al., 2017; da Silva et al., 2010; Borsetto et al., 2017; Charalampaki et al., 2008; Christian et al., 2018; Clark et al., 2010; David et al., 2020; Draf and Schick, 2007; Felisati et al., 2008; Meco and Oberascher, 2004; Flynn et al., 2020; Landeiro et al., 2004; Lund et al., 2000; Placantonakis et al., 2007; Raza et al., 2016; White et al., 2003; Schick et al., 2001; Missale et al., 2022; Xie et al., 2022; Jolly et al., 2022; Radabaugh et al., 2021; Mayer et al., 2021; Jolly et al., 2021; Bubshait and Almomen, 2021; Albaharna et al., 2021; Sharma et al., 2020; Leong et al., 2021; Ali and Raja, 2020; Viera-Artiles et al., 2021; Russo et al., 2022; Ferroli et al., 2021; Yoneoka et al., 2020; Aljawi and Shkoukani, 2023; Benedict et al., 2023; Sheth et al., 2022), ICG (Shahein et al., 2018; Yano et al., 2016; Yokoyama et al., 2016; Cho et al., 2020; Hide et al., 2015; Amano et al., 2019; Hachem et al., 2018; Jeon et al., 2019; Kerr et al., 2017; Geltzeiler et al., 2018; Komatsu et al., 2017; Simal Julian et al., 2016; Sandow et al., 2015; Moy et al., 2019; Lee et al., 2018a; Riley et al., 2019; Fong Ng et al., 2021; Abdelwahab et al., 2020; Ueba et al., 2013; Shaikh et al., 2022), and somatostatin receptor ligands (Linsler et al., 2019, 2021).

### 3.1 5-Aminolevulinic acid

5-ALA is a naturally occurring prodrug that serves as a precursor in heme synthesis. It is metabolized in various tumors and results in accumulation of PpIX (Stummer and Suero, 2017; Hadjipanayis and Stummer, 2019b; Eljamel, 2015; Hadjipanayis et al., 2019; Proskynitopoulos et al., 2020; Suero Molina et al., 2021a). Intraoperatively, 5-ALA-induced fluorescence can be visualized with a microscope equipped with a suitable light source and filter block (e.g., BLUE400 Carl Zeiss Meditec AG, Oberkochen, Germany; FL400 Leica Microsystems, Wetzlar, Germany, or similar), by endoscopes (i.e., D-Light, Karl Storz, Tuttlingen, Germany), surgical loupes (Reveal FGS, Designs for Vision, Inc., USA) (Suero Molina et al., 2021b) or exoscopes (i.e., Orbeye®, Olympus, Tokyo, Japan; Aesculap Aeos®, B. Braun, Tuttlingen, Germany; Synaptive Medical Toronto, Ontario, Canada). 5-ALA excitation maximum is found at 405 nm, presenting the highest fluorescence at 634 nm with other peaks at 620 nm and 704 nm^115^Suero Molina et al. (2023).

The clinical study that led to 5-ALA regulatory approval by the European Medicines Agency (EMA) has become a landmark article and one of the most cited papers in modern neurosurgery (Stummer et al., 2006). A key aspect of the application of this technology was the ability to show a significant difference in the extent of resection as well as progression-free survival when compared to patients who had undergone comparable surgeries under white light microscopy. Published in 2006 but including patients operated on in the late 90s and early 2000s, this article demonstrated a new capability to delineate glioma tissue from adjacent non-diseased brain tissue. Intraoperative 5-ALA-induced visible fluorescence has subsequently also been reported in skull base tumors, i.e., meningioma, chordoma, and inverted papilloma (Cornelius et al., 2019; Goryaynov et al., 2019; Soleman et al., 2013).

### 3.2 Fluorescein

The use of fluorescein sodium salt (FNa) needs to be reported in this context since it harbors both potential advantages as well as risks: On the one hand, it may be a valuable fluorescent dye in detecting cerebrospinal fluid (CSF) leaks, but on the other hand, it carries the potential for rare but serious complications (Table 2).

**(Table 2):** Advantages, Disadvantages, and Limitations of Fluorophores in the resection of skull base tumors.

Fluorophore	Advantages	Disadvantages	Limitations
5-ALA	When fluorescence occurs, it can guide resection. Potential for photodynamic therapy in fluorescent tumors.	Not all tumors exhibit accumulation of 5-ALA or demonstrate fluorescence.	Applied dose, and time-dependency has not been thoroughly examined in skull base diseases.
Fluorescein	Tumor delineation in meningiomas. Video-angiography to identify patency of vessels.	Unspecific. BBB breakdown marker. Fluorescence should be critically interpreted.	Fluorescence requires careful interpretation. Applied dose and time dependency need to be further standardized.
ICG	Assesses patency of vessels. Identifies vascular anatomical landmarks in endoscopic endonasal surgery. Evaluates real-time vascularity in vascularized flaps. Reduced interference from hemoglobin in the NIR	Unspecific. Fluorescence should be critically interpreted.	Applied dose and time dependency need to be further standardized. In the second-window technique, camera gain can be adjusted to enhance exposure when fluorescence signals are weak, though this may also elevate background noise.

BBB = blood-brain barrier, 5-ALA = 5-aminolevulinic acid, ICG = indocyanine green

Of note, FNa was the first fluorescent dye applied in neurosurgery as early as 1947 (Moore, 1947). In this first clinical use by Moore et al., diffuse propagation of FNa within the peritumoral edema was noticed (Moore, 1947). It then fell into oblivion for some time but regained attention and popularity in the 1990s after Zeiss introduced a new filter system, YELLOW560, in the OPMI Pentero microscope (Carl Zeiss Meditec AG; Oberkochen, Germany). By now, other commercially available microscopes have introduced similar visualization filter systems (e.g., FL560, Leica Microsystems). The most robust FNa fluorescence is generated at an excitation light wavelength of 480 nm, emitting a maximum at 525 nm (Zhang et al., 2014). However, white light illumination during endoscopy facilitates clear visualization of FNa fluorescence (Raza et al., 2016). One article reported a statistically significant difference in FNa fluorescence when comparing tumor tissues, scar, and pituitary gland parenchyma during adenoma resection using color spectrophotometric analysis (Romano-Feinholz et al., 2019), and another report presented a similar experience (Bongetta et al., 2021). However, most of the literature dealing with the application of FNa in skull base surgery focuses on vascularity or different topics, as indicated below.

### 3.3 Indocyanine green (ICG)

The FDA initially approved indocyanine green (ICG) in the context of cardiovascular and liver function diagnosis in 1959. In neurosurgery, ICG has also been predominantly used during neurovascular procedures to assess the patency of vessels (Raabe et al., 2003; Hachem et al., 2018; Simal Julian et al., 2016; Bruneau et al., 2013).

Contrary to 5-ALA-induced fluorescence, which occurs in the visible spectrum, ICG is a dye with optical properties near the infrared range (NIR; 700–1000 nm excitation and emission wavelengths) (Lee et al., 2018a). Visualization of the NIR spectrum offers advantages such as reduced scattering of emitted light, minimal disturbance from autofluorescence, and superior penetration of incident photons (Hadjipanayis and Stummer, 2019a). Also, reduced interference from hemoglobin in the NIR range might be of advantage (Table 2). These characteristics overcome some limitations of those fluorophores that fluoresce within the visible range, e.g., they do not interfere with hemoglobin autofluorescence (Hadjipanayis and Stummer, 2019a). ICG demonstrates a peak excitation wavelength of 780 nm and emits within the range of 805–825 nm^32^. The ICG fluorescence can be visualized using standard optical microscopes (e.g., FLOW800, Carl Zeiss Meditec AG, Oberkochen, Germany; Leica Microsystems, Germany), exoscopes (i.e., Orbeye, Olympus, Tokyo, Japan), or endoscopes (Karl Storz, Tuttlingen, Germany; VisionSense^tm^ iridium, Medtronic). Of interest is the fact that there is already FDA approval for perfusion imaging with ICG in plastic and reconstructive surgery cases to assess flap viability in real time.

## 4 Discussion

### 4.1 5-Aminolevulinic acid

#### 4.1.1 5-ALA in endoscopic endonasal skull base surgery

A recent multicenter retrospective study evaluated 28 patients treated for skull base lesions (Micko et al., 2020), including pituitary neuroendocrine tumors (PitNET) (n = 15, 54%), meningiomas (n = 4, 14%), craniopharyngiomas (n = 3, 11%), Rathke's cleft cysts (n = 2, 7%), and cases of plasmacytoma, esthesioneuroblastoma, and sinonasal squamous cell carcinoma. Notably, no chordoma was included in this series. The authors concluded that, except for meningioma, 5-ALA facilitated the resection of these pathologies (Micko et al., 2020). This article is a significant attempt to extend the established use of the dye, however, several methodic concerns in this retrospective evaluation must be considered. Firstly, and most relevant to further usage, it is questionable if all included tumor pathologies exhibit the same kinetics in accumulating protoporphyrin IX (Recinos, 2020). In an article by Kaneko et al., real-time measurements in human tumor samples with the help of spectroscopy were performed in samples of malignant glioma (Kaneko et al., 2019). The authors found that the fluorescence peak in human tissues occurred about 7–8 h after administration, in contrast to the conventional 6-h time window recommended, which was based on *in-vitro* experiments before 5-ALA regulatory approval (Kaneko et al., 2021). In the abovementioned study, 5-ALA was administered 2.5–4 h before surgery, which might be too close to the time of surgery. Furthermore, bleaching could have induced a significant bias in this study since strong endoscope LED lighting may cause rapid fluorescence bleaching. Thus, a diligent time-dependency analysis of fluorescence kinetics in individual skull base pathologies is needed.

#### 4.1.2 5-ALA and pituitary neuroendocrine tumors

Although typically benign, PitNET can cause severe complications through invasion or destruction of local structures, or hormonal and metabolic disturbances. Some PitNETs pose challenges in visualization, leading to incomplete resection of tumors, which is often due to poor visualization of infiltrating tumor tissue into adjacent spaces, such as the cavernous sinus, the sphenoid bone, or the suprasellar cistern. Furthermore, predominantly in “MR-negative” cases, microadenoma localization is often challenging. Thus, implementing a real-time aid to better visualize PitNet could address these limitations.

Uptake of 5-ALA and accumulation of PpIX in different PitNET cell lines has been demonstrated (Neumann et al., 2016; Nemes et al., 2016). In a study by Eljamel et al. the efficacy of 5-ALA-fluorescence (administered 3 h before surgery) to detect pituitary adenoma was assessed using two different devices: an endoscopic-mounted photo diagnostic filter and a laser-based probe for intraoperative spectrometry. The study demonstrated that fluoroscopically enhanced endoscopy with 5-ALA exhibited a sensitivity of 80.8% and specificity of 75%. In comparison, intraoperative spectrometry had a sensitivity of 95.5% and specificity of 100% (Eljamel et al., 2009). However, other investigators have reported meager fluorescence rates of only 8% of patients (1/12) (Marbacher et al., 2014), although the low sample size hampered this observation. Further understanding of factors predicting intraoperative fluorescence in PitNETs (e.g., tumor size, vascularization, cell density, tumor cell type, etc.) and the most appropriate time and method for fluorescence detection technology is thus necessary.

#### 4.1.3 5-ALA and skull base meningiomas

20–30% of meningiomas are located in the skull base (Nanda and Vannemreddy, 2008). These tumors are challenging to treat due to their proximity to cranial nerves and vessels and their deep location. Beyond that, it can be difficult to identify brain and bone invasion, dura tails’ extent, infiltration of the falx cerebri, and/or optic canal invasion/skull base foramens (Lee et al., 2018a). Furthermore, scar and tumor tissue can be macroscopically similar in the recurrent setting or after radiotherapy.

Meningiomas have been observed to fluoresce after oral 5-ALA administration (Cornelius et al., 2014, 2017a, 2019; Goryaynov et al., 2019; Turcotte et al., 2020; Coluccia et al., 2010; Cornelius et al., 2019). A statistically significant correlation between fluorescence intensity and the histologically specified WHO grade has been reported (Cornelius et al., 2014). Fluorescence rates, specificity, and sensitivity were reported as high in featured publications, although mostly in small series (Bekelis et al., 2011; Cornelius et al., 2014; Potapov et al., 2016; Coluccia et al., 2010). The integration of imaging with positron emission tomography and 5-ALA guidance helped resect a recurrent skull base meningioma (Cornelius et al., 2013a), illustrating how different metabolic imaging tools may complement each other.

Bone invasion has been discussed as a potential factor for recurrence in meningiomas (Abdelzaher et al., 2011). Hyperostosis, primarily caused by tumor bone invasion (Pieper et al., 1999), is associated with tumor recurrence, morbidity, and mortality (Della Puppa et al., 2014; Riley et al., 2019). However, achieving clear macroscopic delineation between invaded and non-invaded bone presents challenges, as MRI signal changes, even beyond hyperostosis, do not necessarily correlate with tumor invasion (Fathalla et al., 2020). Della Puppa et al. described the advantage of fluorescence guidance in identifying bone infiltration and avoiding excessive drilling of healthy bone in meningioma (Della Puppa and Scienza, 2013; Della Puppa et al., 2014). With 5-ALA-guided fluorescence, the reported sensitivity for tumor depiction was 89.06%, with a specificity of 100% in a cohort of 12 patients. The positive and negative predictive values were reported as 100% and 82.93% (95% CI 71.41%–94.45%), respectively.

In recurrent tumor cases, 5-ALA guidance enhanced the precision of resection by distinguishing between the tumor and both adjacent brain tissue and nasal mucosa during endonasal resections (Cornelius et al., 2013a). Advancements in FGS for meningiomas have led Cornelius et al. to suggest supplementing the Simpson grading with additional information about the fluorescence status (Cornelius and Slotty, 2014).

Intraoperative quantitative assessment of meningioma by spectroscopy was first described in 2011 by Bekelis et al. (2011). In a subsequent small series by Potapov et al., spectroscopy-aided navigation assisted in finding small meningioma remnants, tumor infiltration in hyperostotic bone, and even an infiltrated adventitia in an M2 branch (Potapov et al., 2016). Also, in endoscopically-assisted skull base surgery cases, a specially dedicated filter system was described for visualizing 5-ALA fluorescence in tissue of anterior skull base meningioma (Cornelius et al., 2019). The authors concluded that this surgical adjunct was feasible and helpful.

Although skull base meningiomas tend to be of low WHO grade (Cornelius et al., 2013b), and meningioma tissue is generally easily identified under white-light microscopy, there are still some advantages in using FGS, especially in tumors of higher grades (WHO° 2 and 3) (Cornelius et al., 2014): identification of local infiltration of the brain or arachnoid, their attachment to surrounding veins or arteries, their invasion of bone and dura, and differentiating them from scar or irradiated tissues in recurrent situations which can be very challenging.

An international multicenter prospective study (NXDC-MEN-301) (Stummer et al., 2022) was initiated to determine the clinical utility and safety of such use in primary and recurrent meningiomas and to answer many current questions in managing these tumors. The outcome of this study is expected in 2025.

#### 4.1.4 5-ALA and chordomas

Chordomas, primary malignant tumors of notochord origin, are generally located on both extremities of the spine: the skull base, craniocervical junction, or at the sacrum and spine (Yasuda et al., 2012; Chibbaro et al., 2014; Passeri et al., 2022; Yadav et al., 2023). Exceptionally, they may arise outside the axial skeleton (Evans et al., 2016; Lee et al., 2021). Chordomas pose unique treatment challenges. They exhibit invasive and destructive behavior and are highly resistant to chemotherapy and radiotherapy. In vitro studies exploring various adjunctive treatments have shown that chordoma cells are susceptible to 5-ALA PDT. An experimental study demonstrated time-dependent 5-ALA uptake after incubation in chordoma cell lines (U-CH2) and the feasibility of experimental PDT (Cornelius et al., 2017b; Gull et al., 2021). PpIX accumulation was higher after 6 h than after 4 h, which aligns with time-dependency studies done in high-grade glioma (Kaneko et al., 2019).

Furthermore, cell destruction was correlated to treatment by 5-ALA-based PDT, suggesting uptake and metabolization by chordoma cells. Personal experience provided insight into 5-ALA-based fluorescence in a case of microscopic surgery of recurrent chordoma (unpublished personal data of JFC). Even though these results might not yet be generalized to in vivo applications, these preliminary observations are promising. Consequently, future research is needed to analyze real-time in vivo kinetics in chordoma tumors.

#### 4.1.5 Miscellaneous

Furthermore, 5-ALA-induced fluorescence application can be of great use in non-skull base tumors where conventional skull base approaches are employed, i.e., craniopharyngiomas, complex tumors with intricate locations near the pituitary gland or metastatic lesions originating from distant primary tumors.

Craniopharyngiomas pose unique surgical challenges due to their proximity and adherence to vital structures. At the same time, many metastatic lesions cannot be radically resected, whereas others often necessitate meticulous resection to alleviate symptoms and improve patient prognosis.

Our literature research identified a singular case of a skull base schwannoma exhibiting 5-ALA-induced fluorescence, which is typically not reported from nerve-sheath tumors (Goryaynov et al., 2019). However, this technique is not a common practice in the surgical removal of these tumors and may yield valuable results.

By further investigating the utility of 5-ALA for previously unexplored territories of skull base pathology, we may open new research avenues for promising diagnostic strategies and alternative adjunctive treatments, such as Photodynamic therapy (PDT).

PDT is currently under study in the context of second-line therapy for recurrent gliomas (Schipmann et al., 2020). During PDT, free radicals and reactive oxygen particles lead to cytotoxic damage of tumor cells after PpIX excitation at 635 nm (Henderson and Dougherty, 1992). Two articles (Neumann et al., 2016; Nemes et al., 2016) explored the effect of PDT in different cell lines from pituitary adenomas, including GH3, AtT-20, and human cell cultures. Here, in all cell lines and the primary cell culture, there was evidence of 5-ALA uptake and effective accumulation of PpIX. Interestingly, in human cell cultures, a toxic effect was observed only with increased 5-ALA concentrations (Neumann et al., 2016). PDT could be explored as an option for skull base tumors but has not yet been studied.

### 4.2 Fluorescein

#### 4.2.1 Fluorescein and CSF leaks

CSF rhinorrhea due to a skull base defect can be challenging to treat. β_2_-transferrin and beta-trace protein are reliable biochemical markers with high specificity and sensitivity for detecting CSF, often avoiding unnecessary invasive procedures, such as, e.g., CT cisternogram (Borsetto et al., 2017; Lescuyer et al., 2012). However, once CSF leakage has been confirmed, surgery is usually needed. Intrathecal Fluorescein (IF) injection has been used to localize skull base defects precisely, thus ensuring integrity of repairs (Borsetto et al., 2017; Charalampaki et al., 2008; Draf and Schick, 2007; White et al., 2003; Schick et al., 2001; Missale et al., 2022; Xie et al., 2022; Jolly et al., 2022; Radabaugh et al., 2021; Albaharna et al., 2021; Aljawi and Shkoukani, 2023; Benedict et al., 2023; Sheth et al., 2022).

**Fig. 1:**
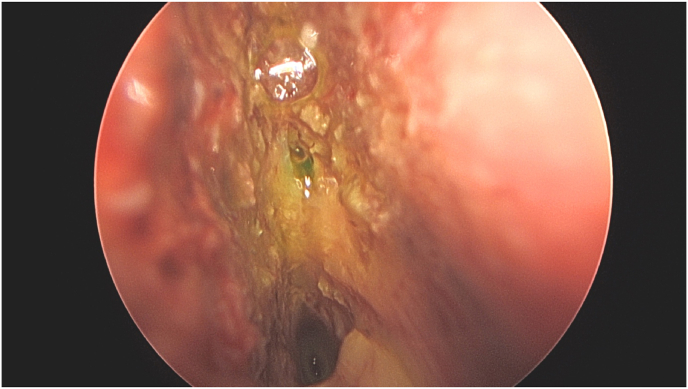
– Endoscopic endonasal view of intrathecal applied fluorescein demonstrating an anterior skull base defect in a young female patient with spontaneous rhinoliquorrhea.

First described by Kirchner and Proud in the 1960s (Kirchner and Proud, 1960), IF has become part of the clinical routine in the surgical management of CSF leaks in numerous centers despite its off-label use. The current summary product characteristics (SPC) of Fluorescein states that it should not be administered intrathecally. A survey performed in 1978 with the members of the American Association of Neurological Surgeons (AANS) already displayed the range of potential complications was already established, i.e., transient paresis, numbness, seizures, and cranial nerve deficits, with doses applied from 0.1 to 5 ml of 5% Fluorescein injected after being diluted with 0–10 ml of CSF (Anari et al., 2007; Placantonakis et al., 2007; Moseley et al., 1978). One case report noted persistent paraplegia after an IF injection of 20 mg (2% saline mixture) (Mayer et al., 2021). A delayed absence seizure 8 h after injection has also been reported as a complication after intrathecal fluorescein injection (Anari et al., 2007). As an alternative, a topical application (5%) of Fluorescein has also been discussed, demonstrating similar sensitivity after intrathecal application (Xie et al., 2022; Yoneoka et al., 2020). In both scenarios, a change in the color of Fluorescein from yellow to green with appropriate excitation light has been reported, indicating the presence of CSF (Bubshait and Almomen, 2021) (Fig. 1).

A dye dilution (1:10,000,000) and a dose of about 25 mg i.v. has proven sufficient for clear visualization with the endoscope (Draf and Schick, 2007; Jolly et al., 2022) (Table 3). Current reports indicate the safety of low-dose IF, such as 0.25 ml of 10% Fluorescein solution dissolved in 10 ml CSF (25 mg), or even 1 ml of 5% concentration diluted in 9 ml CSF (50 mg) (Banu et al., 2014; Zhang et al., 2018; Clark et al., 2010; Felisati et al., 2008; Placantonakis et al., 2007; Raza et al., 2016; Keerl et al., 2004). Complications seem to occur in less than 0.1% of patients (Keerl et al., 2004). Importantly, no Fluorescein drug with a 5% concentration has been approved by the FDA or the EMA. Every major complication reported in the literature after intrathecal administration seems related to incorrect dosage, mainly when using over 100 mg and certainly >500 mg of total Fluorescein (Felisati et al., 2008). The clinical experience with Fluorescein-guided CSF leak detection involves more than 2000 reported patients, enhancing the clinical experience of using Fluorescein for these indications (Felisati et al., 2008; Meco and Oberascher, 2004; Landeiro et al., 2004; Raza et al., 2016; Jolly et al., 2021, 2022). In an extensive series of 419 patients, sensitivity and specificity of IF were 92.9% and 100%, respectively, in this setting. The positive and negative predictive values were 100% and 88.8%, indicating IF as a specific and sensitive tool for intraoperative CSF leak diagnosis and repair (Raza et al., 2016).

**(Table 3):** Dosing, administration routes, and potential side effects of fluorescent dyes commonly used in skull base surgery.

Fluorescent Dye	Dose/Administration	Time of Administration	Potential Side Effects
5-Aminolevulinic Acid (5-ALA)	20 mg/kg; Oral administration.	Administered 2.5–4 h prior to surgery.	- Photosensitivity (e.g., skin irritation, rash) lasting up to 48 h. - Gastrointestinal discomfort (e.g., nausea, vomiting). - Temporary elevation of liver enzymes.
Indocyanine Green (ICG)	5–25 mg; Intravenous administration.	Administered 16–30 h preoperatively or intraoperatively.	- Mild allergic reactions (e.g., rash, pruritus). - Rare risk of anaphylactic shock. - Potential cardiovascular effects (e.g., hypotension, tachycardia).
Fluorescein	Intravenous: 3–10 mg/kg; typically 5 mg/kg dose. Intrathecal: 0.25 ml (25 mg) of 10% or 1 ml (50 mg) of 5% concentration diluted in cerebrospinal fluid.	Administered intraoperatively.	- Yellow discoloration of skin and urine. - Nausea, vomiting. - Rare but severe allergic reactions, including anaphylaxis. - Seizures (at higher doses).

Even in the pediatric population, the clinical usefulness of IF has been reported. In a small series by Locatelli et al. IF was useful as a diagnostic and intraoperative tool in 11 of 12 cases to assess the location of the CSF leak and confirm closure (Locatelli et al., 2006). IF has also helped visualize CSF leaks in cadaveric specimens, i.e., in the context of simulation and training of skull base repair techniques (Christian et al., 2018).

Authors of a recent systematic review advised against introducing IF to clinical practice (Albaharna et al., 2021). By now, the Food and Drug Administration (FDA) has neither prohibited nor indicated the off-label intrathecal use of Fluorescein (Felisati et al., 2008). Therefore, extreme caution is needed since the medico-legal environment has not been determined.

#### 4.2.2 Fluorescein and meningiomas

Identification of tumor margins and use in video-angiography have been described as possible applications of Fluorescein in meningioma surgery (Akcakaya et al., 2017). Akcakaya et al. observed in a cohort of 30 patients homogenous enhancement in most meningiomas; however, low-intensity and diffuse heterogeneous enhancement patterns were also described (Akcakaya et al., 2017). In this small series, Fluorescein could reveal tumor infiltration in bone tissue, which provided guidance when deciding on margins for drilling. Furthermore, during video-angiography, Fluorescein proved valuable in assessing the patency of vessels and perforating arteries, cortical drainage veins, and the microvasculature of the pial surface of cranial nerves after tumor removal (Akcakaya et al., 2017; Ferroli et al., 2021). Other small series reported a “good” visualization of meningioma tissue, aiding in the dissection and preservation of neural tissues (da Silva et al., 2010; da Silva et al., 2014a; da Silva et al., 2014b) since cranial nerve enhancement was not observed. However, a comparison to the gold standard of “white light microscopy” has not been undertaken yet, and the actual clinical value of this adjunct measure remains unclear.

As briefly mentioned above: The applied dose of the fluorophore needs to be standardized, and the time dependency of maximal tissue concentration needs to be thoroughly studied in humans. Both parameters can strongly influence visual fluorescence (Suero Molina and Stummer, 2017; Stummer, 2016). In available published reports, the applied dose was 1000 mg^55,147^ or 2–4 mg/kg body weight (Akcakaya et al., 2017) (Table 4), and Fluorescein was administered either immediately before assessment or after the induction of anesthesia with modern series, most frequently opting for the latter.

**(Table 4):** Experimental fluorescent dyes in skull base surgery.

Fluorophore	Potential Utility
Bevacizumab-800CW	Binds and neutralizes all isoforms of human vascular endothelial growth factor A (VEGF-A). It is under research to detect PitNET tissue during endoscopic transsphenoidal surgery.
Folate Receptor Near-Infrared Imaging	Marks folate in non-functioning pituitary adenomas and meningiomas that overexpress folate receptor alpha with OTL38.
Somatostatin Receptor Ligands	FAM-TOC evaluated in primary cell cultures from patients harboring meningiomas. Demonstrated strong fluorescence after incubation with FAM-TOC.
Tozuleristide	Combination of tumor-targeting peptide chlorotoxin and NIR fluorophore ICG.

ICG = indocyanine green, NIR = near-infrared, PitNET = Pituitary Neuroendocrine Tumor.

Fluorescein remains a non-specific marker for breakdown of the blood-brain barrier (BBB) that extravasates similarly to vasogenic edema, resulting in staining of normal (non-neoplastic) brain (Suero Molina and Stummer, 2017).

The number of existing published series currently lacks studies with randomization and those involving multiple centers. Therefore, more data is needed to accurately assess the specificity, sensitivity, and clinical benefits of this fluorophore in meningioma surgery.

#### 4.2.3 Fluorescein and PitNET

The treatment of PAs, particularly aggressive or recurrent ones that invade the cavernous sinus, requires innovative surgical techniques to enhance outcomes.

In this context, Romano-Feinholz et al. describe a pilot study on the use of hybrid fluorescein-guided surgery for resection of pituitary adenomas, FNa, a fluorophore in use for over 50 years in various medical conditions (Romano-Feinholz et al., 2019). The study explores the feasibility and safety of FNa in guiding tumor resection, utilizing an endonasal endoscopic approach combined with microscopic techniques under a special YELLOW 560 filter for enhanced visualization. The study included 15 patients with different types of PAs, showing no FNa-related complications and demonstrating a significant difference in fluorescence among tumor, gland, and scar tissue, which could facilitate more effective and safer tumor resections.

This research signifies the first of its kind to assess the use of FNa in pituitary adenoma surgeries, highlighting its potential benefits in achieving better surgical outcomes, particularly for hormone-producing and recurrent tumors. The technique's safety and feasibility were established, with findings suggesting it could also reduce the learning curve in pituitary adenoma surgery. However, the study acknowledges its limitations, including a small cohort and a short follow-up period, suggesting the need for further trials to validate these preliminary findings.

#### 4.2.4 Fluorescein for droplet staining in endonasal surgery

Recently, several articles described the utility of nasal application of FNa as a visual marker for droplet formation and “risk of contamination” and intraoperative safety assessments during endonasal surgery in the context of the current SARS-CoV-2 pandemic (David et al., 2020; Leong et al., 2021; Ali and Raja, 2020; Viera-Artiles et al., 2021; Russo et al., 2022). Activating drills or other rotating instrumentation outside the nose caused gross droplet contamination (Sharma et al., 2020).

### 4.3 Indocyanine green

#### 4.3.1 ICG in endoscopic endonasal surgery

**Fig. 2:**
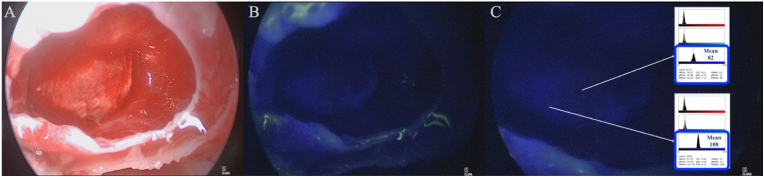
A case of ICG fluorescence after endoscopic endonasal resection of intrasellar PitNET. A single dose 25 mg was given upon completing sphenoidal step of the procedure. In **(A)** endoscopic view after surgical resection shows the pituitary gland dislocated laterally and posteriorly. In **(B)** the ICG endoscopic view show differences in the fluorescence of the gland caused by different degrees of compression by the tumor. In **(C)** measurement of ICG fluorescence representing when the maximum and minimum blue color values were reached in the pituitary gland, with attached screenshots from the measurements generated using ImageJ software. (For interpretation of the references to color in this figure legend, the reader is referred to the Web version of this article.)

ICG has been used to identify vascular anatomical landmarks in endoscopic endonasal surgery. Applied doses ranged from 6.5 to 25 mg as a single dose ICG (Litvack et al., 2012; Verstegen et al., 2016; Lee and Lee, 2022; Shahein et al., 2020; Hide et al., 2015; Amano et al., 2019) (Fig. 2) and were administered intravenously immediately prior to evaluation of the surgical field (Litvack et al., 2012; Verstegen et al., 2016; Shahein et al., 2018). ICG is usually diluted in 10 ml sterile water or 0.9% NaCl. In the first published series (n = 38), Hide et al. demonstrated the application of ICG in identifying the internal carotid artery (ICA) and smaller vessels. In this study, the indication was for differentiating adenoma tissue from the pituitary gland or distinguishing the pituitary stalk from craniopharyngioma in real time (Hide et al., 2015). A strong fluorescence could be observed in the ICA whilst fluorescence of the cavernous sinus lagged for several few seconds (Hide et al., 2015). Differences in tissue enhancement were measured by retrospectively evaluating the observed fluorescence intensity and the time-dependence of fluorescence of different structures. The authors advocated ICG to increase the spatial resolution under the endoscope and helped with orientation in moments that necessitate confirming the patency of smaller vessels. Another clinical series of 33 patients focused on analyzing the characteristics of dye distribution (Amano et al., 2019). Unfortunately, most figures in the respective publications are not as informative as they could be, with obscure visualization of presented landmarks.

Nevertheless, the dye is a powerful surgical adjunct where rise and transit time (Holling et al., 2013) were analyzed. Both articles demonstrated that PitNET enhanced later and for a shorter duration than non-neoplastic pituitary tissue (Hide et al., 2015; Amano et al., 2019). Similarly, a "delayed" window displaying ICG up to 90 min post-administration facilitated real-time visualization of PitNET during endoscopic endonasal surgery. (Muto et al., 2023). These authors reported an approximately 6 times stronger fluorescence signal in the normal pituitary gland at 90 min when compared to PitNETs (Muto et al., 2023). To facilitate the interpretation of these images, Shahein et al. created an instructive flowchart to ease ICG signal interpretation in PitNETs (Shahein et al., 2020). These heterogeneous results suggest the need for further research before clinical application.

The “second window” of suitable ICG fluorescence was described after applying high doses of ICG at about 16–30 h prior to surgery and subsequently observing selective enhancement of tumor tisse (Jeon et al., 2019). This technique has been successfully explored in malignant glioma and meningiomas (Lee et al., 2018a; Zeh et al., 2017). The term “second window” was introduced to distinguish this technique from the traditional ICG video angiography, in which fluorescence is visualized a few seconds after intravenous injection of a 25 mg bolus of ICG. In a small cohort of 15 PitNET patients, an ICG bolus was administered at 5 mg/kg body weight; the ICG bolus was administered 24 h before surgery. In this study, sensitivity was calculated as slightly higher but at lower specificity when data were compared to observations made with white light for tumor identification (Jeon et al., 2019). Another study by Cho et al. (2020) applied ICG at a 2.5 mg/kg body weight 24 before surgery. It demonstrated its potential use to visualize the pituitary stalk during skull base surgery, enabling surgeons to confidently identify and preserve the stalk even in complex cases of significantly distorted anatomy.

**Fig. 3:**
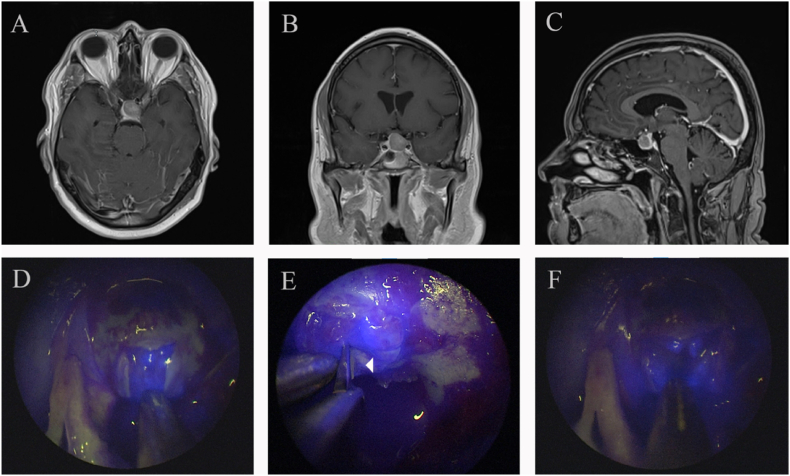
(**A)** axial, (**B)** coronal, and (**C)** sagittal MRI post-contrast images of a pituitary macroadenoma where the gland is pushed to the right side and is covering the sellar face. **(D)** shows opening of the dura and underlying hyperfluorescent pituitary gland. **(E)** shows sectioning of the gland to reveal the underlying hypofluorescentwadenoma. **(F)** shows a cruciate incision of the pituitary gland and the underlying hypofluorescent adenoma.

Identifying pituitary adenoma tissue is not demanding in most routine and de novo cases; however, in the setting of recurrence or fibrous PitNETs, especially after radiotherapy or after long-term Dopamine agonist treatment (Menucci et al., 2011), this technique might provide additional safety to the resection of these tumors (Inoue et al., 2021) (Fig. 3).

#### 4.3.2 ICG and meningiomas

Using the same abovementioned method of “second-window” assessments, other authors have explored the value of this technique in meningioma surgery (Lee et al., 2018a). In an article by Lee et al. ICG was administered 18–28 h before surgery at an intravenous dose of 5 mg/kg body weight in a cohort of 18 patients (mean age 55 years, range 20–74) (Lee et al., 2018a). This article demonstrated how time dependency is essential: The few patients that did not exhibit significant intraoperative fluorescence (n = 4, 22%) displayed instead an “inversed” fluorescence pattern, in which the adjacent brain had a higher NIR signal than the tumor tissue when imaged 21 h after dye administration. Even though an intensity plateau of fluorescent signal was observed between 6 and 72 h in a rodent model of intracranial brain tumors, in-vivo real-time fluorescence measurements at different time points have not been systematically studied (Hadjipanayis and Stummer, 2019a). In the article by Lee et al. none of the assessed parameters, such as age, tumor size, perifocal edema, tumor location, and histopathological features, approached statistical significance as predictors for fluorescence via logistic regression, except for the time from administration to imaging (p < 0.05) (Lee et al., 2018a). This clearly illustrates the relevance of considering time dependency when applying fluorescent dyes, even though the visualization window appears rather broad. For each tumor type, understanding the duration of fluorescence and the timing of its peak is paramount for optimizing the use of these dyes in clinical applications.

Another essential application for ICG was described by Ueba et al. highlighting the utility of ICG for transdural visualization to localize venous sinus, surface veins, tumor tissue, and pial supply (Ueba et al., 2013).

#### 4.3.3 ICG-video angiography (ICGva)

Super-selective intra-arterial chemotherapy for recurrent skull base cancer has been reported to be effective by increasing drug delivery, resulting in higher concentrations in tumor tissue. A report by Yokoyama et al. demonstrated the efficacy of ICG video angiography in assisting super-selective intra-arterial chemotherapy for malignant lesions. In a small series (n = 7), ICGva successfully visualized the tumor's blood supply and feeding arteries in all analyzed cases, addressing challenges sometimes encountered with other imaging tools like computed tomography angiography (CTA), especially in cases involving dental implants (Yokoyama et al., 2016).

Fong et al. reported visualizing a cavernous hemangioma in the lateral orbital apex with ICG after 90 s of intravenous application. The authors declared that using ICG was helpful during transorbital surgery when incising the periorbita directly at the lesion and in dissecting the tumor from surrounding tissue (Fong Ng et al., 2021).

Another case report demonstrated further use of ICGva when resecting a vestibular schwannoma via a translabyrinthine approach (Hachem et al., 2018). In this setting, ICGva revealed obstructed flow in the sigmoid sinus, later confirmed as thrombosis on a postoperative CT scan. This early detection allowed for prompt initiation of treatment. Even though no systematic evaluation of this indication has yet been undertaken, this may be of significant potential future use.

#### 4.3.4 ICG and vascularized pericranial and nasoseptal flap

The vascularized nasoseptal flap has been the “reconstructive workhorse” in endoscopic endonasal surgery over the past two decades (Geltzeiler et al., 2018). Thus, unrecognized impairment of its blood supply could lead to a higher rate of flap failure and postoperative CSF leak. To this end, ICGva has been explored in several studies (Shahein et al., 2018; Kerr et al., 2017; Geltzeiler et al., 2018; Komatsu et al., 2017). In these reports, an ICG bolus of 12.5–25 mg was applied intravenously (Kerr et al., 2017; Geltzeiler et al., 2018), and the approximate time to visualize ICG fluorescence was 20–25s with the endoscope (Kerr et al., 2017; Geltzeiler et al., 2018) and 2.4 min with the microscope (Sandow et al., 2015).

Geltzeiler et al. demonstrated a high correlation between intraoperative fluorescence and postoperative MRI enhancement and the incidence of postoperative flap necrosis rate. Interestingly, only 53% of patients in this study showed an enhancement of both the body and the pedicle of the flap, with one developing a subsequent CSF leak. Overall, two patients with enhancement of the pedicle alone or no enhancement at all presented with flap necrosis. In this study, 6/38 patients (15.8%) had a postoperative CSF leak. No statistically significant difference between ICG fluorescence and the rate of CSF leak was reported. However, the cohort in this study was rather small (Geltzeiler et al., 2018). In another series, four out of five patients demonstrated homogenous fluorescence enhancement throughout the nasoseptal flap, while a heterogeneous fluorescence pattern could only be observed in one patient. No postoperative CSF leaks were reported (Kerr et al., 2017).

Other publications demonstrated the intraoperative use of ICGva during endonasal surgery (Shahein et al., 2018; Simal Julian et al., 2016). ICG was useful here to monitor vessel-patency and to localize the ICA. ICGva has opened new diagnostic monitoring options when analyzing viability of microvascular free tissue transfer, as published in a case of a radial forearm free flap covering a tissue defect at the craniocervical junction (Moy et al., 2019).

**Fig. 4:**
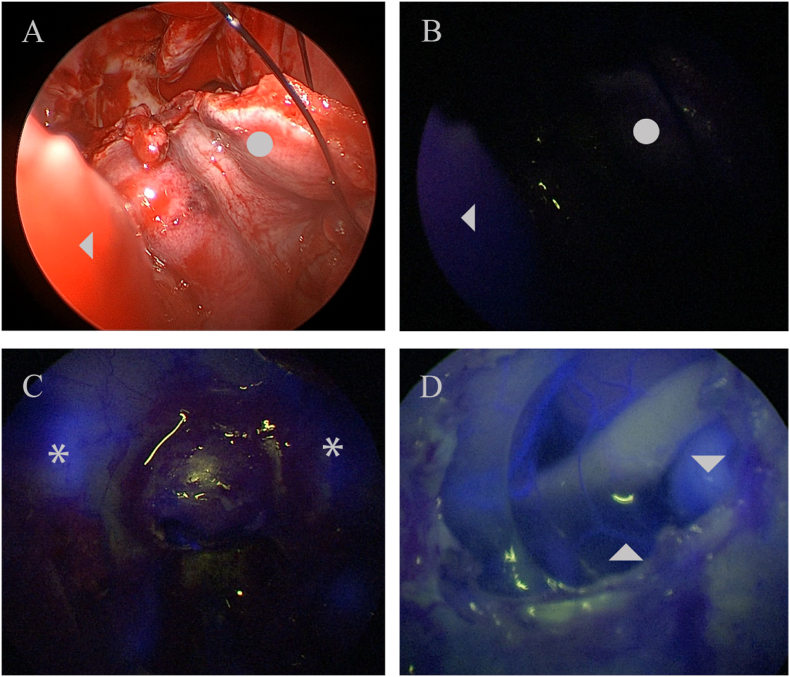
A case of a recurrent chordoma in which a nasoseptal flap has been reutilized. In **(A)** normal endoscopic mode shows the proximal portion^◄^ of the flap and the distal portion^●^of the flap. In **(B)** shows the ICG endoscopic view where the distal portion^●^is hypofluorescent in comparison to the proximal portion^◄^. **(C)** shows a case of pituitary microadenoma with the beginning of the ICG fluorescence in the Internal carotid artery * on both sides. **(D)** shows a case of resected tuberculum sellae meningioma and the underlying optic nerve, left Internal carotid artery▼, and the superior hypophyseal artery ^▲^.

In skull base surgery, intraoperative ICG fluorescence has been correlated with postoperative MRI contrast enhancement of the nasal flap (Fig. 4). However, it has not been correlated with flap necrosis or failure of reconstruction, presumably due to the low number of reported cases (Shaikh et al., 2022). More extensive case series are needed to correlate skull base repair success rates and intraoperative ICGva parameters.

ICG has been applied based on a variety of indications for skull base surgery. Still, differences in timing and dosing are further complicated by the fact that various imaging software processes were used. It appears that ICG is non-specific. An overlay technique can be selectively applied; this somehow complicates the depth perception in 2D fields assessed with the endoscope.

Pericranial flaps (PCF) are regularly used to reconstruct complex skull base dural defects. Perfusion, as in vascularization, has been a critical factor in demonstrating the efficacy of vascularized PCF for closure. ICGva assisted in evaluating real-time vascularity in PCF and, therefore, assessed flap viability (Komatsu et al., 2017; Abdelwahab et al., 2020; Shaikh et al., 2022). In their assessment by Yano et al., anteriorly-based PCF showed better vascularity than laterally-based PCF (Yano et al., 2016). With ICG imaging as a surgical adjunct, tissue perfusion was significantly reduced in smoking, older, and overweight patients compared to individuals without these risk factors, due to compromised vascularity. Preoperative radiation of the tumor bed did not lead to worse vascularity in the analyzed cohort of PCF cases. However, no radiation dose, modality, or details of treatment timing were presented. The standard amount of ICG applied was 0.1 mg/kg body weight.

#### 4.3.5 Time dependency and dosage of different fluorophores

Dosage and time dependency are critical considerations when employing fluorescent dyes in surgical procedures. Time dependency refers to the time period required for fluorophores to accumulate and fluoresce within the target tissue. It has become evident that the various fluorophores exhibit varying dosage- and time dependencies. For instance, 5-ALA is administered orally at 20 mg/kg body weight approximately 4 h before anesthesia induction (Stummer et al., 2006). However, this dosage was tested in the context of malignant gliomas and may not be universally applicable to other tumor types. Time dependency can vary significantly according to the target tissue and tumor type since 5-ALA typically takes around 4 h to accumulate in malignant glioma, while suitable concentrations may not be reached in meningiomas or pituitary adenomas due to differences in metabolisms.

Dosage and time dependency can also significantly impact fluorophore efficacy. Insufficient dosage may result in inadequate fluorophore accumulation within the target tissue, producing a weak fluorescence signal. Conversely, excessive dosage may produce side effects or induce toxicity. It is crucial to carefully consider fluorophore dosage and time dependency, recognizing that these factors have yet to be thoroughly studied in the various applications discussed in this article.

### 4.4 Outlook

#### 4.4.1 Non-invasive CSF detection

In a recent report, a shortwave infrared (SWIR)-optimized rigid endoscope was utilized to identify CSF leaks by exploiting the similar absorption properties of CSF and water (Klein et al., 2023). The authors present a promising outlook for a potential alternative to the current invasive diagnostic methods. Such novel tools utilizing intrinsic chemical properties of relevant compounds would eliminate the need for invasive exploratory procedures or the application of specific contrast agents.

### 4.5 Experimental fluorophores

#### 4.5.1 Folate receptor near-infrared imaging

Folate receptor near-infrared imaging involves the use of OTL38 (On Target Laboratories, West Lafayette, Indiana, USA), a folate analog conjugated to a cyanine dye, as a biomarker for tumor tissues that overexpress folate receptor alpha (Cho et al., 2018, 2020). Cho et al. demonstrated that OTL38 provided high specificity for detecting non-functioning pituitary adenomas, with significant differentiation in the signal-to-background ratio between adenomas that overexpress folate receptor alpha and those that do not (Cho et al., 2018). Other studies found OTL38 to be highly specific, especially in non-functioning adenomas (Amano et al., 2019; Lee et al., 2018b). In meningiomas, Lee et al. (2018a) demonstrated highly sensitive detection of tumor tissue, with a sensitivity as high as 96.4% and a specificity of 38.9%, a positive predictive value of 71.1%, and a negative predictive value of 87.5% was calculated for tumor detection. A limitation is the lack of preoperative knowledge of folate receptor expression, which may impair sensitivity (Jarmula et al., 2022) (Table 4).

#### 4.5.2 Somatostatin receptor ligands

In an ex-vivo setting, the fluorescent dye FAM-TOC (5,6-Carboxyfluoresceine-Tyr3-Octreotide) was evaluated in 24 primary cell cultures from patients harboring WHO I (n = 16), WHO II (n = 6) and WHO III (n = 2) meningiomas (Linsler et al., 2019). In this study, 22/24 (91.7%) demonstrated strong and 2/24 (8,3%) weak fluorescence after incubation with FAM-TOC (Linsler et al., 2019). Even though this article lacked a validated control group (which means that it does not necessarily tell us whether fluorescence can distinguish tumor tissue or dura tail from scar tissue or adjacent normal brain), such results are promising and should be further studied. In a follow-up article by the same group, primary meningioma cell culture samples from meningiomas were implanted in nude mice after transfection with FAM-TOC (Linsler et al., 2021). The authors conclude that this technique is of value, as it enabled better tumor margins visualization and allowed, as confirmed by autopsy, complete resection in all animals (Linsler et al., 2021).

#### 4.5.3 Tumor-targeting molecule and Tozuleristide (NIR)

tumor-targeting molecule: The combination of the tumor-targeting peptide chlorotoxin and the NIR fluorophore ICG has been explored in distinct tumors (Hadjipanayis and Stummer, 2019a). Chlorotoxin is believed to have an affinity to tumors and is non-toxic to humans. Its utility has been demonstrated in other cancer model systems and is subject of current research.

#### 4.5.4 Bevacizumab-800CW

is a fluorescent tracer molecule that binds and neutralizes all isoforms of human vascular endothelial growth factor A (VEGF-A) (Vergeer et al., 2021). The DEPARTURE trial represents the first exploration of bevacizumab-800CW used to detect PitNet tissue during endoscopic transsphenoidal surgery (Vergeer et al., 2021). The study utilizes multidiameter single-fiber reflectance and single-fiber fluorescence spectroscopy to quantify fluorescence intensities in vivo and ex vivo. The study includes a diverse group of PitNET tumors with varying VEGF-A expression levels. As a phase 1 exploratory trial, the data collected may not directly impact currently available treatment but aims to assess the feasibility of bevacizumab-800CW-mediated fluorescence for PitNET visualization (Vergeer et al., 2021). This trial has not been completed at the time of submission of this review.

#### 4.5.5 Limitations of this review

While FGS presents a promising avenue for enhancing the precision of tumor resections and identifying vital structures during neurosurgical procedures, its effectiveness is curtailed by various factors that necessitate further exploration and improvement. Despite its success in glioma surgery, the application of FGS in skull base surgery remains underexplored and poorly understood. This gap in knowledge and application indicates a pressing need for more extensive research and literature to validate and expand its use. Moreover, the ongoing development of quantitative and semi-quantitative methods for assessing fluorescence intensity and quality points to the limitations of the qualitative nature of fluorescence assessment, suggesting an area ripe for technological advancement.

A critical evaluation of the existing literature reveals a predominance of early-stage studies, such as proof of concept, case reports, and cohort studies, with a notable lack of rigorous case-control or matched studies, thus limiting the strength of recommendations.

Another point worth mentioning is the challenge of blood-brain barrier (BBB) penetration by many fluorescent dyes, which hampers their efficacy in targeting tumor tissues within the central nervous system without prior disruption of the BBB. This can affect the utility of FGS in precisely delineating tumor margins. Additionally, not knowing the variability in the timing and metabolic processing of dyes like 5-ALA by different tumors affects its specificity and sensitivity.

Additionally, the literature addressing the use of fluorescein for investigating cerebrospinal fluid (CSF) leaks primarily pertains to endoscopic endonasal surgery. The discussions on the utility of 5-ALA in assessing bony invasion combine cases of cranial vault meningiomas with skull base tumors, where the decision for bony resection often relates more to the surgical approach rather than the extent of bony invasion itself. It is noteworthy that achieving “Simpson 1” resection in skull base meningiomas, which includes bone removal, besides when feasible through an endonasal approach, is rarely the primary objective of this type of surgery.

While FGS holds significant potential for improving outcomes in neurosurgery, its current limitations underscore the need for continued research, technological innovation, and a broader compilation of high-quality evidence.

## 5 Conclusion

This article summarizes current techniques, discusses controversies, and presents an extensive overview of possible applications and indications for fluorescence technology in skull base pathologies. It is meant to discuss the current state of the art and provide encouragement and orientation for future research endeavors. Fluorescence guidance emerges as a promising surgical adjunct for treating skull base pathologies, particularly in maximizing tumor resection and identifying and repairing CSF leaks. Despite the research discussed in this article, there is still limited evidence available. Therefore, further studies are needed.

## Funding

Open Acess funding enabled and organized by Project DEAL.

## Declaration of competing interest

The authors declare the following financial interests/personal relationships which may be considered as potential competing interests:

Eric Suero Molina reports a relationship with Carl Zeiss Meditec AG that includes: funding grants. Walter Stummer has received speaker and consultant fees from Medac, Carl Zeiss Meditec AG, Leica Microsystems, Photonamic, and NXDC and funding grants from Carl Zeiss Meditec AG. If there are other authors, they declare that they have no known competing financial interests or personal relationships that could have appeared to influence the work reported in this paper.
